# Aerosol emissions from wind instruments: effects of performer age, sex, sound pressure level, and bell covers

**DOI:** 10.1038/s41598-022-15530-x

**Published:** 2022-07-04

**Authors:** John Volckens, Kristen M. Good, Dan Goble, Nicholas Good, Joshua P. Keller, Amy Keisling, Christian L’Orange, Emily Morton, Rebecca Phillips, Ky Tanner

**Affiliations:** 1 Department of Mechanical Engineering, Colorado State University, Fort Collins, CO 80523 USA; 2 Department of Environmental and Radiological Health Sciences, Colorado State University, Fort Collins, CO USA; 3 School of Music, Theatre, and Dance, Colorado State University, Fort Collins, CO USA; 4 Department of Statistics, Colorado State University, Fort Collins, CO USA; 5 Division of Disease Control and Public Health Response, Colorado Department of Public Health and Environment, Denver, CO USA

**Keywords:** Environmental sciences, Engineering, Infectious diseases

## Abstract

Aerosol emissions from wind instruments are a suspected route of transmission for airborne infectious diseases, such as SARS-CoV-2. We evaluated aerosol number emissions (from 0.25 to 35.15 μm) from 81 volunteer performers of both sexes and varied age (12 to 63 years) while playing wind instruments (bassoon, clarinet, flute, French horn, oboe, piccolo, saxophone, trombone, trumpet, and tuba) or singing. Measured emissions spanned more than two orders of magnitude, ranging in rate from < 8 to 1,815 particles s^−1^, with brass instruments, on average, producing 191% (95% CI 81–367%) more aerosol than woodwinds. Being male was associated with a 70% increase in emissions (vs. female; 95% CI 9–166%). Each 1 dBA increase in sound pressure level was associated with a 28% increase (95% CI 10–40%) in emissions from brass instruments; sound pressure level was not associated with woodwind emissions. Age was not a significant predictor of emissions. The use of bell covers reduced aerosol emissions from three brass instruments tested (trombone, tuba, and trumpet), with average reductions ranging from 53 to 73%, but not for the two woodwind instruments tested (oboe and clarinet). Results from this work can facilitate infectious disease risk management for the performing arts.

## Introduction

The COVID-19 pandemic has raised awareness for a need to improve scientific understanding surrounding the modes and mechanisms that govern the spread of airborne infectious diseases^1^. Recent research has demonstrated that human respiratory aerosol (airborne particles generated from the human respiratory tract, typically with sizes spanning < 0.1 to 100 μm in diameter) plays a prominent role in SARS-CoV-2 transmission^1–5^.

Performing arts activities, for example, singing and playing wind instruments, are considered high-risk scenarios for airborne infectious disease spread, as these activities often take place indoors and may involve large gatherings and close proximity, for extended periods of time, of performers and audience members. Despite the elevated risk, there is limited data about whether and how performing arts activities play a role in the transmission of airborne infectious disease (such as SARS-CoV-2), though evidence, to date, has implicated the vocal performing arts. In March 2020, for example, 53 of the 61 individuals from a choir in Skagit County, Washington, USA became infected with COVID-19 following a single practice, with detailed contact tracing^6^ and modeling^7^ supporting an airborne (and aerosol-based) mode of transmission. Although the playing of wind instruments represents a plausible route for aerosol-based transmission, such activities have not yet been implicated in outbreaks or specific transmission events related to COVID-19. Further, more research is needed to evaluate whether source-control technologies like bell covers are effective at reducing the potential for disease spread, as has been suggested previously^8,9^.

Respiratory aerosol is generated through actions such as breathing^10–12^, talking^10,11,13–16^, singing^10,15,16^, coughing^11,17,18^, and sneezing^17,19^. These actions originate within various regions of the respiratory tract^11^ and subsequent aerosol emissions tend to vary in size^16,20^, concentration^15,16,20^, and relative composition^21,22^ (the latter related to varying compositions of pulmonary lung lining fluid, tracheobronchial and nasal mucosa, and saliva). For example, breathing produces particles from the pulmonary region of the lung following collapse/reopening of terminal bronchioles^20,23,24^, vocalization produces particles following abduction of the vocal folds under subglottal pressure^20^, and talking combines the former modes with the release of larger particles following articulation of the tongue and lips^20^. Research on human vocal emissions has reported that singing (vs. breathing and talking)^10,15,16,25^, being male (vs. female)^15^, adult (vs. minor)^15,25^, and vocalizing at higher sound pressure (i.e., voice volume) levels^13,15^, were all correlated with increased emissions of respiratory aerosol.

The playing of wind instruments involves controlled breathing and oral contact/articulation with a mouthpiece, during which time both expired air and saliva flow into the instrument. Therefore, in similar fashion to vocalization, one could hypothesize that playing of wind instruments could lead to emission of respiratory aerosols from breathing and subsequent aerosolization of saliva from the vibrating instrument. Recent work supports this hypothesis^8,9,26,27^, but published literature is somewhat contradictory regarding the magnitude of emissions from different instruments. There is also uncertainty regarding sex and age effects and whether available mitigation measures (i.e., bell covers) are effective at reducing emissions and subsequent exposure risks.

The goal of this work, therefore, was to characterize aerosol emissions from wind instruments from a large panel of performers of varying age and sex. Multi-level models were developed to evaluate differences in these emissions as a function of instrument and demographic variables, including instrument class, type, and sound pressure level, participant age and sex, and the form of music being played. The mixed models include a random intercept for each participant, which accounts for correlation in repeated measures from the same individual and allows for assessment of within- and between-participant variation. A secondary goal was to evaluate the effect of bell covers on mitigating aerosol emissions from select wind instruments.

## Results

### Aerosol emission factors

A total of 81 participants completed the measurement protocol, spanning ages from 12 to 63 years at enrollment. Participant age and sex (assigned at birth) demographics are shown in Figure S2. Approximately half (n = 41) of the participants were minors and 42% (n = 34) were female.

**Figure 1:**
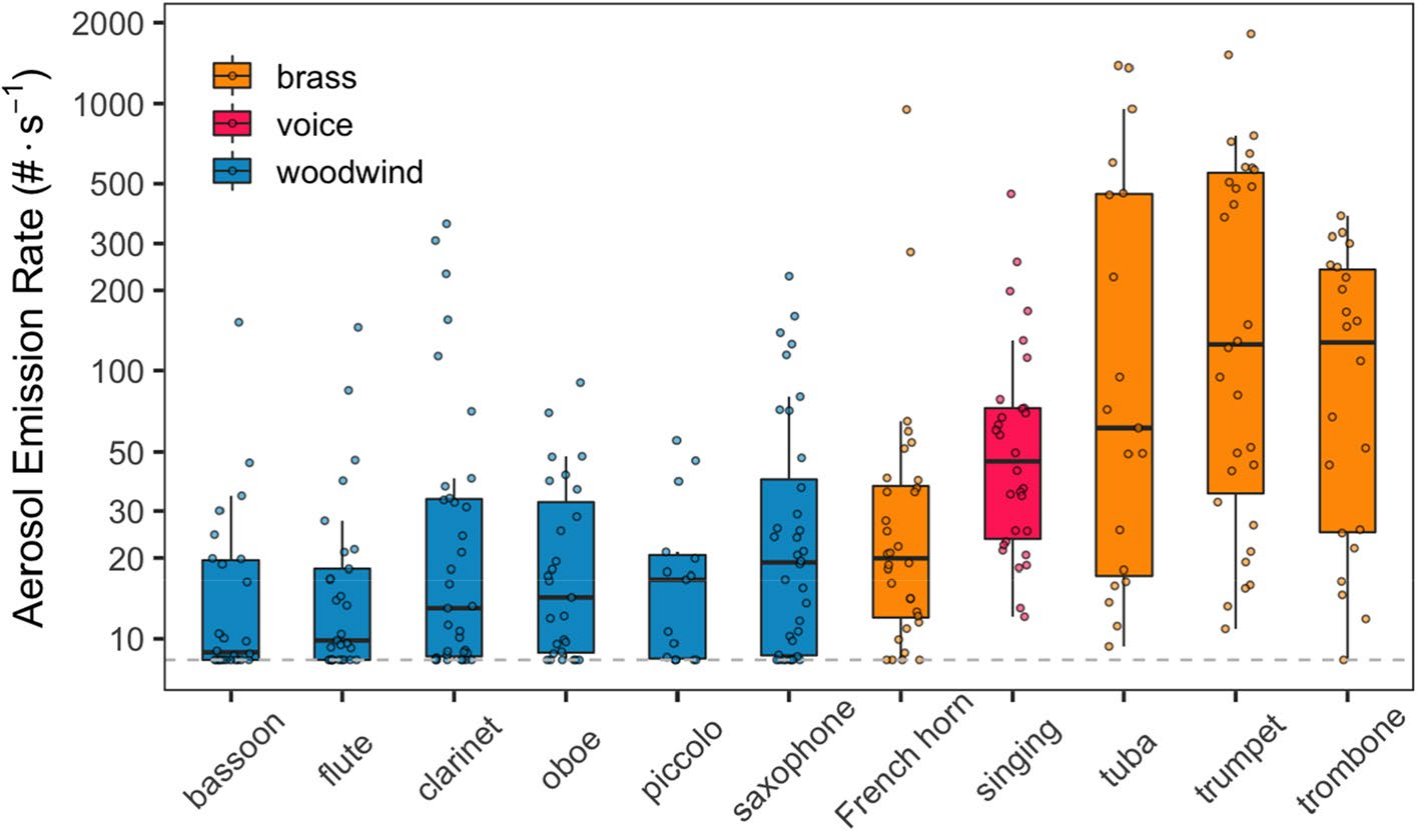
Boxplots of aerosol number emission rates (0.25–35.15 μm size range) by instrument class and type, including vocalists. Instruments are ordered by median emission rate with participant data as open circles; boxes delineate the inter-quartile range (IQR) and whiskers extend to 1.5⋅IQR or the data minimum. The dashed horizontal line represents a method quantification limit (8.3 particles s^−1^). Data are background corrected.

Shown in Fig. 1 are distributions of emission factors by instrument type (and colored by instrument class), as measured by the optical particle counter (OPC). Aerosol emission rates from wind instruments varied over several order of magnitude, ranging from < 8 to 1,815 particles s^−1^. This logarithmic variation is evident both between and within a given instrument type, as can be seen for the tuba, for which measured emission rates ranged from 10 to 1,400 particles s^−1^ across participants.

Results from the mixed model indicate that 24% of the emissions variability is due to the fixed effects of sex, age, and class of instrument, 39% is due to additional participant variation (i.e., beyond age and sex), with the remaining 37% of emissions variation unexplained by our model. As shown in Figure S3, the variability in emissions from one participant to the next is considerable, although many individuals produced emissions that varied by a factor of 10 or more across their maneuvers. The type of maneuver (scales, selection, freestyle) was not a significant predictor of emissions (*p* = 0.5; descriptive results shown in Table S4).

A clear distinction is evident between emissions from brass and woodwind instruments (Fig. 1 and Figure S4), with the model estimating that brass instruments, on average, emit 191% more particles than woodwinds (95% CI 81–367%). Singing emissions, which tended to overlap with brass instruments, were also significantly higher than woodwinds (179%, CI 60–386%). Median emission rates within class varied by as much as a factor of 2 (e.g., flute vs. saxophone, French horn vs. tuba). Descriptive data tables of emissions by instrument type are provided in the online supplement (Tables S1–S3).

Average particle size distributions are shown in Figure S7 for each instrument type. The shape of the particle size distributions was similar across instrument types with a primary mode at 0.4 μm and secondary mode at ~ 2 μm. These size data are consistent with prior reports^26,27^.

### Demographic differences

**Figure 2:**
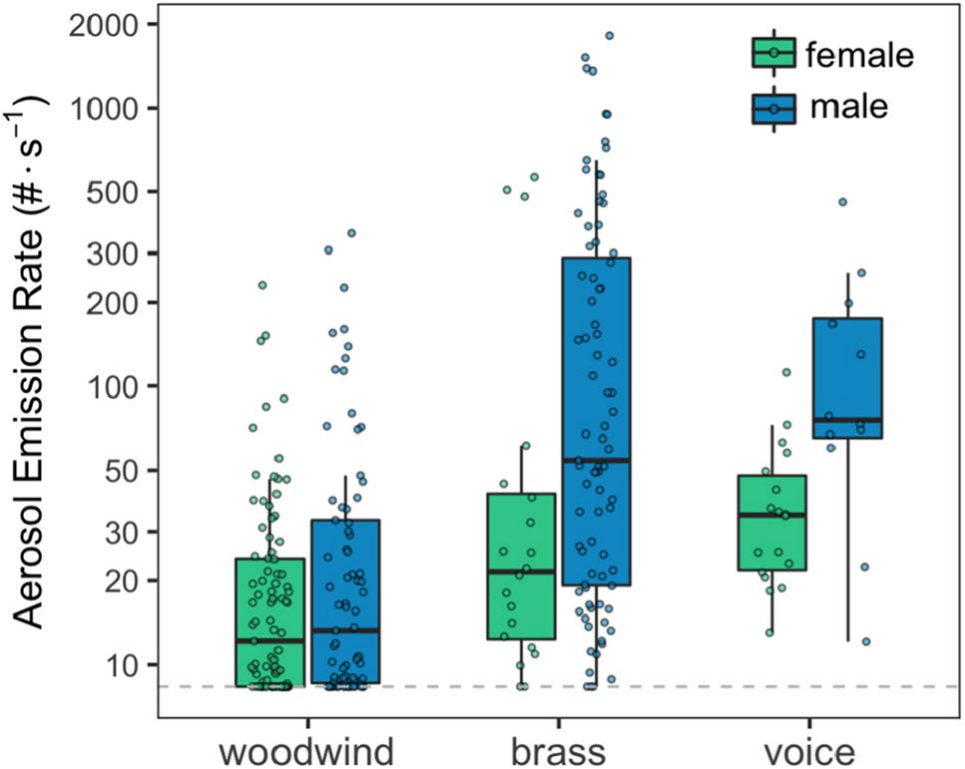
Boxplots of aerosol number emission rates (0.25–35.15 μm size range) by instrument class and participant sex (assigned at birth). Box limits delineate the inter-quartile range (IQR) with median values at center; whiskers extend to 1.5⋅IQR or the data minimum. The dashed horizontal line represents a method quantification limit (8.3 particles per second). All data are background corrected.

Participant sex was a significant predictor of emissions (*p* = 0.017). As can be seen in Fig. 2, male brass and male vocal performers tend to emit more particles than female woodwind, brass, and vocal performers. On average, the mixed model estimates that males emit 70% (CI 9–166%) more particles than females. Sex differences are even more pronounced across instrument classes; for example, when the interaction between sex and instrument class is considered, male brass players emit 408% (CI 116–1093%) and male vocalists emit 356% (CI 159–702%) more particles than female woodwind players. Emission rates are not significantly different between males and females within the brass and woodwind instrument classes (*p* = 0.47), however, male singers emit significantly more aerosol than female singers (141%; CI 16–406), which is consistent with our prior work^15^. Participant age was not a significant predictor of emissions in models that included sex and instrument class as covariates (*p* = 0.25; Figure S5).

### Effect of sound pressure level

**Figure 3:**
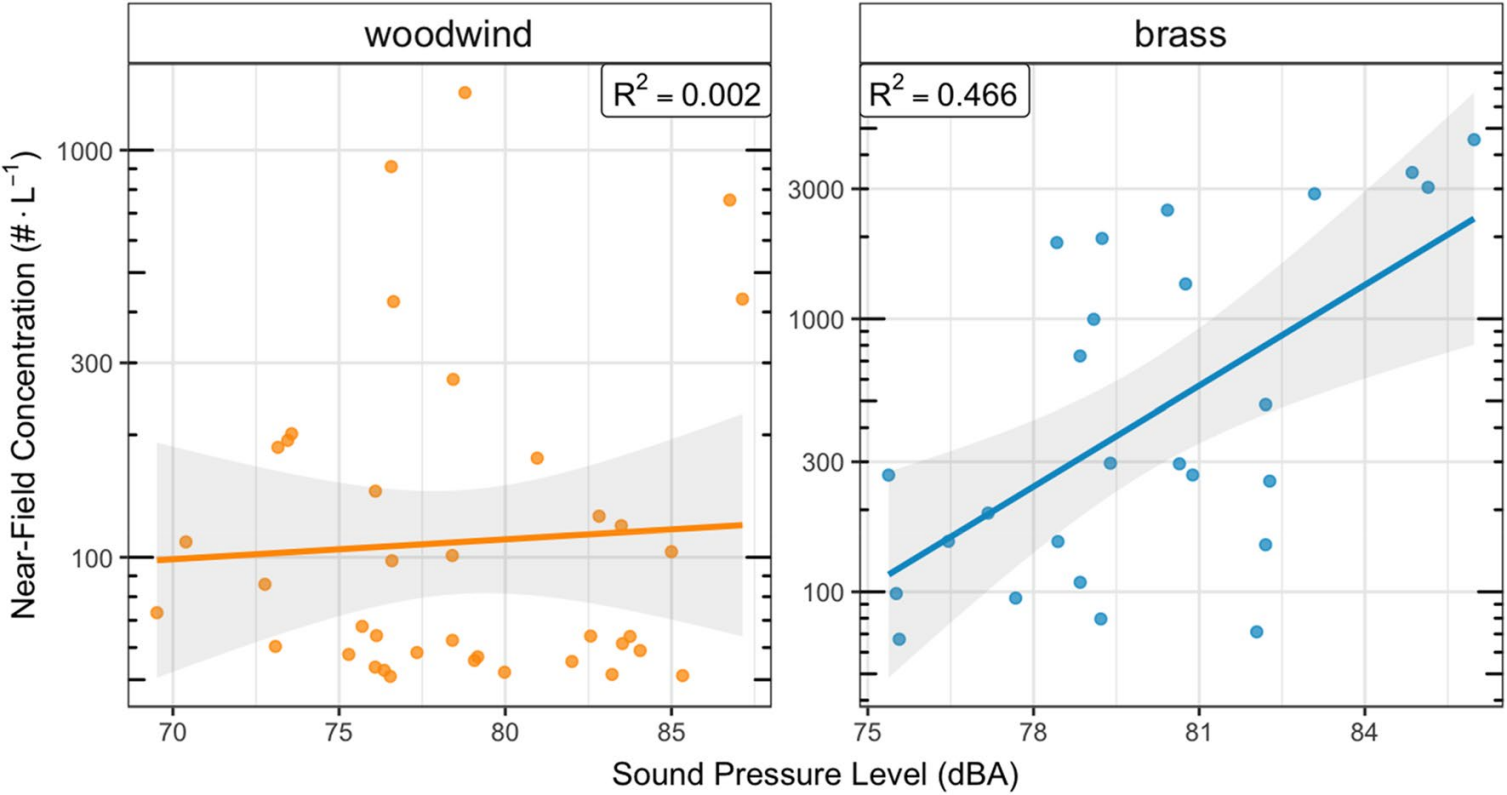
Correlation between instrument sound pressure level and aerosol number emission rates (0.25–35.15 μm size range), stratified by instrument class. All data are background corrected; correlations consider only measurement data above method detection limit.

Prior research suggests that sound pressure levels are correlated with aerosol emissions from vocalization^13,15^ and may be correlated with aerosol emission from instruments^26,27^. We find evidence of a statistically significant correlation between sound pressure levels and aerosol emissions for brass instruments (r^2^ = 0.357), but not for woodwinds (r^2^ = 0.003), as shown in Fig. 3. Results from a mixed model including sound pressure level as a fixed effect suggest that each 1 dBA higher noise level produces 28% (CI 10–40%) more aerosol number emissions from brass instruments. For woodwinds, the change in aerosol emissions per unit dBA is only 2.5% (CI − 3 to 8%). We note that sound pressure levels were not correlated with sex (i.e., males did not tend to vocalize or play instruments at higher sound pressure levels than females).

### Effect of bell covers

**Figure 4:**
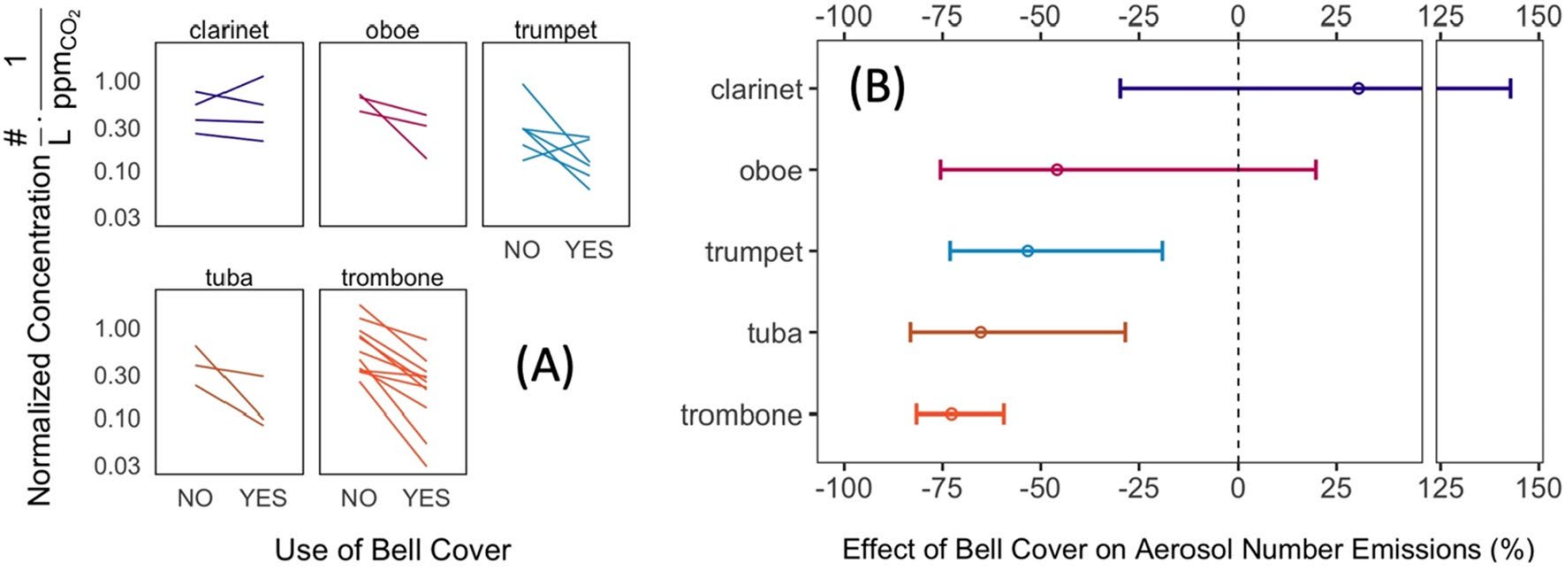
Effect of bell covers on reducing aerosol number emissions (0.25–35.15 μm size range) from instruments. (**A**) Participant-specific changes in CO_2_-normalized aerosol number concentration with bell covers (each line represents a pair of measurements for a participant with and without a bell cover). (**B**) Average percent reduction in emissions with 95% confidence intervals. Data are background corrected and restricted to instruments with n ≥ 3 measurement pairs.

The use of bell covers produced a statistically significant reduction in aerosol emissions for 3 of 5 instruments tested, all of which were brass, as shown in Fig. 4. The estimated effect of bell covers for the two woodwind instruments tested (oboe and clarinet) was not significantly different from zero (Fig. 4B). Bell covers results are not reported for bassoon, flute, French horn, piccolo, and saxophone due to low sample size (i.e., less than 3 measurements per instrument).

Sensitivity analyses using near-field concentration data (that were not normalized to CO_2_ emissions) produced similar (statistically significant) conclusions for brass instruments, though use of near-field concentration data (i.e., without CO_2_ normalization) tended to overestimate the effectiveness of bell covers (Figure S9). For example, not accounting for CO_2_ losses resulted in an overestimate of bell cover effectiveness for brass instruments from anywhere between 6 and 27%, based on Figure S9. For the clarinet, CO_2_ normalization attenuated the perceived effect of the bell cover altogether (Figure S9).

## Discussion

A strength of our study is the large sample size (n = 81 volunteers), which allowed us to examine the effects of age and sex demographics on wind instrument emissions. To our knowledge, this is the largest panel study examining aerosol emissions from wind instruments to date. The large sample size allows us to better understand inter-individual differences, as well as provide greater statistical power to explore how differences in performer demographics impact emissions, which results in our data being more generalizable to a wider population of performers. We note however, that even our sample size lacked adequate power to test for sex and age differences within individual instrument types, given the large variability in emissions seen within (Figure S3) and between (Fig. 1) participants.

Our results suggest that the sex of the performer, the class of instrument being played, and the sound pressure level of the instrument (for brass instruments) are important drivers of aerosol emissions from wind instruments. Additional factors may also contribute to the large amount of within-person variation in wind instrument emissions seen here. One hypothesis is that saliva, which flows into the mouthpiece over time, might need time to build up sufficiently to become aerosolized. Salivary flow rates also show inter-person^28,29^ and diurnal variation^30^. We did not quantify salivary production rates among participants, so this hypothesis remains untested; however, we did not observe a relationship with emissions over time (i.e., from one maneuver to the next) in our study. Our results also suggest that the type of music being played (i.e., scales vs. self-selection vs. prescribed selection) was not a significant predictor of aerosol emissions. Our measurements were averaged over ~ 4 min period, however, and other reports on second-to-second variations in emissions have shown that changing articulation patterns^26^ and playing styles^8^ can modulate short-term emission rates.

Our emissions data overlap with those of McCarthy et al.^27^ and He et al.^26^, who reported near-field concentrations from a similar set of instruments, albeit among smaller panel sizes than our study (n = 9 and n = 15, respectively). For woodwinds, our results support the conclusion of McCarthy et al.^27^ that “*aerosol number and mass generated during instrument playing…is lower than that associated with singing and speaking at high volume.*” With brass instruments, however, we find that emissions are on par with those for speaking and singing (Fig. 1 and^15^), in contrast to McCarthy et al. Our emissions variability (spanning over 2 orders of magnitude) is also consistent with that of He et al.^26^ However, He et al. characterized the tuba as low-risk relative to human vocal emissions and the oboe as high-risk, which is somewhat contrary to our results. We attribute these discrepancies to a difference in sample size, as our results suggest that person-to-person variability can be dramatic (Figure S3); He et al. tested only 2 participants per instrument and McCarthy et al. tested 9 subjects playing 13 total instruments, whereas we tested 81 subjects playing 10 instruments (n = 7.2 participants per instrument, on average). Given the lognormal variability we observed between participants, it is not surprising that our median instrument emission rates differ from studies with fewer participants, as the variation in emissions from one participant to the next could span a factor of 3 or more (Figure S3).

We find that sound pressure level is positively correlated with aerosol emissions from brass instruments; however, our mixed models suggest that sound pressure level explains some, but not all the effects seen for instrument class and participant sex. McCarthy et al.^27^ reported a correlation between sound pressure levels and aerosol emissions across their panel but did not quantify the magnitude of the effect due to low sample size. Interestingly, He et al.^26^ reported a positive correlation between sound pressure levels and emissions for three woodwind instruments (oboe, clarinet, bassoon) but not for any brass instruments.

The reason(s) behind the increased emissions for male brass players and singers is unknown, though our prior work on vocalization suggests that lung size/capacity, which is higher in males, explains some of the differences in vocal emissions by sex^15^. We see slight increases in CO_2_ mixing ratios from both male vocalists and brass players in our panel (relative to females; Figure S6), but not from male woodwind players. A second potential reason is that males tend to produce more saliva, on average, than females. Inoue et al. reported that the rates of salivary flow and protein secretion were 42% and 73% higher, respectively, in males vs. females^29^. We did not quantify salivary flow rates in our panel; this represents a potential line of future inquiry. We did not assess participant skill level, which could be a factor in emissions; however, we did not observe a statistically significant effect of age on emissions (minors vs. adults *p* = 0.25, Figure S5) and there was a noticeable difference in skill levels between minors and adults in our panel. Most of our adult performers were either professional musicians, volunteer orchestra members, or music majors and most of our minor performers were recreational trainees.

Our results suggest that bell covers can substantially reduce particle emissions from brass instruments, but their effectiveness for woodwind instruments remains uncertain, based on the two instruments tested here. One hypothesis for why bell covers are more effective on brass instruments (vs. woodwinds) is that more airflow tends to exit through the bell of a brass instrument, as compared to a woodwind, for which a varied amount of air typically exits from one or more tone holes, where the bell cover is not present. Such leakage, even in the absence of a bell cover, has been reported previously^8,26^. Bell covers were not uniformly effective across participants (Fig. 4A); we hypothesize that, similar to the use of respirators, masks, and face coverings, a proper fit must be achieved between the cover and the instrument to function as intended. Mask/respirator fit (i.e., leakage around the mask) is a major determinant of effectiveness^31–33^ and we noted during experimentation that not all bell covers produced a tight fit against a given instrument.

Abraham et al. reported a 60% reduction in aerosol emissions from a trumpet when played with a bell cover made from a single layer of filter fabric, which is consistent with the results shown in Fig. 4A^9^. Stockman et al. also reported a 50% reduction in CO_2_-normalized aerosol emissions from a clarinet with a bell cover in place^8^. As shown in Fig. 4A, two of the four clarinet players from our panel showed a reduction in CO_2_-normalized aerosol emissions when using a bell cover, one showed a near zero change, and one showed an increase in CO_2_-normalized emissions. For the participant that had an apparent increase in CO_2_-normalized emissions, this effect was caused by a steep reduction in measured CO_2_ mixing ratios when the bell cover was used (as opposed to an actual increase in near-field aerosol concentrations). This result suggests that the pressure differential imparted by a bell cover is likely to force additional air out through the instrument tone holes, which could potentially attenuate bell cover effectiveness (especially for woodwind instruments). The added pressure differential of a bell cover may also affect sound quality and instrument playability, as reported previously^9^.

Additional strengths of our study include a carefully controlled experimental protocol and the use of mixed models to account for correlation within participants. Our tests were conducted in an isolated, temperature and humidity-controlled cleanroom environment, our data were background corrected, and we employed an isokinetic, constant-volume sampling apparatus, which is a standard, reproducible method for aerosol emissions sampling^34^. The emissions estimates presented here are likely conservative, however, as we may not have captured all the oral emissions (i.e., droplets from the buzzing of lips against a mouthpiece^8^), especially for elongated instruments (e.g., the oboe or trombone) where the mouthpieces were situated farther away from our measurement inlet. However, we report CO_2_-normalized emission factors, which account for some of these potential losses and our overall conclusions remain consistent following CO_2_ normalization, which provides an additional layer of rigor to our results.

We did not assess emissions from young children and whether our results are generalizable outside the age range of our study remains unclear. We chose to limit our participants to ages 12 and older following input from our scientific advisory board, who suggested that this age is when children typically begin to demonstrate sufficient ability to play wind instruments with competence. We also considered age appropriateness for adherence to our study protocol.

Our study did not assess disease transmission risk, nor did we quantify the potential for measured emissions to contain infectious SARS-CoV-2 virions (though we note that such work has been published for vocalization and expired air^35–37^). Rather, this work assumes that emissions of respiratory aerosols correlate with disease transmission risk (i.e., more respiratory emissions would mean greater transmission risk, if the emitting individual was infected). We acknowledge that several other factors play a role in transmission beyond respiratory emission levels. However, our results suggest that singing and playing of wind instruments have the potential to release large amounts of respiratory aerosol, which is consistent with published reports of disease transmission indoors^5^. We note that while brass instruments tended to have higher emissions than woodwinds, all instruments featured one or more “high emitters”, which rivaled (or exceeded) those of singers (Fig. 1). The factors that govern whether a person/performer is a high emitter is a priority for ongoing work. Further, our maneuvers lasted approximately 4 min each and took place in a laboratory setting; we do not know if longer performances would result in different emissions profiles. Additional work is needed to validate our findings in real-world settings.

## Conclusions

The playing of wind instruments produces logarithmic variations in aerosol emissions, with brass instruments emitting more aerosol than woodwinds. Being male and playing at higher sound pressure levels is associated with increased emissions from brass instruments. The variability in emissions spans several orders of magnitude, both within a given instrument type and within a given performer. This variability in emissions presents a challenge for mitigating exposure risks in performing arts settings, as our results suggest that no one single factor is a dominant driver of emissions. However, we do show that bell covers are effective at reducing aerosol emissions from brass instruments, which tend to emit particles at higher rates than woodwinds. Taken together, our results suggest that a layered intervention approach is likely required (masking/bell covers, ventilation, distancing, etc.) to protect from infectious aerosol disease transmission in the performing arts, in the absence of widespread immunity among performers and audience members.

## Methods

### Study subjects

Healthy adult performers, aged 18 years and older, and minor performers, aged 12–18, were recruited to participate in the study. All participants provided written informed consent, or assent in the case of minors (along with informed consent from the parent/guardian), following US regulatory guidelines for research on human subjects (experimental protocols were approved by the Colorado State University Institutional Review Board, approval #20-10174H). We recruited adults and minors (aged 12–17) in roughly equal proportion and in groups of 6–8 performers for each of the following instruments: clarinet, bassoon, flute, oboe, piccolo, saxophone (alto and tenor), French horn, trumpet, trombone, and tuba. In addition, we recruited 16 vocalists (half professional/adult, half minors) to characterize singing emissions. These combinations (7 participants per 10 instruments and 16 singers) gave a target panel size of n = 86. Recruitment included both males and females to evaluate the effect of sex (male or female; assigned at birth) on emissions.

Participants brought their own instruments to the test facility (described below) along with a personal mask/face covering. For safety reasons, participants were excluded from the study if they were actively experiencing any symptoms of COVID-19, had a prior diagnosis of COVID-19 during the previous month, or had a known exposure to someone who had COVID-19 in the previous 14 days (consistent with state and local quarantine protocols at the time of the study).

Each participant completed a series of maneuvers specific to their specialty and ability level, during a ~ 2-h measurement session. These maneuvers included the playing of *scales,* a *prescribed repertoire* that was provided to participants at least two weeks prior to the measurement session [*selection*], a *self-selected repertoire* at the discretion of each participant [*freestyle*], and two generic vocal maneuvers [*talking* and *singing*]*.* This paper is focused on aerosol emissions from instrumentalists and trained singers while performing scales, prescribed selections, and self-selected repertoires (results of the generic vocal maneuvers have been published previously^15^). Each maneuver was repeated continuously over a period of four minutes, during which aerosol emissions were measured. Participants wore lint-free bodysuits and lint-free hair nets (polypropylene disposable coveralls, McMaster-Carr, IL) within the facility to minimize particle shedding from their clothing and hair, respectively. Between each maneuver, a background measurement was taken while participants wore their personal face covering for at least two minutes and sat quietly, ~ 2 m away from the aerosol collection instruments.

### Measurements

Participants undertook the maneuvers inside a 3.45 m × 2.8 m × 2.45 m environmental chamber^15,38^ ventilated with HEPA-filtered air. Chamber airflow was controlled (~ 8.5 air changes per hour) and environmental conditions (temperature, humidity) inside the chamber were logged along with all measurement data using LabVIEW (National Instruments, TX, version 21.0, https://www.ni.com/en-us/shop/labview.html) instrument control and data acquisition software. A constant-volume sampling apparatus (10 L min^−1^ total flow rate) was used to capture aerosol emissions directly downstream of the instrument bell or the participant’s mouth (Figure S1). The sampling apparatus was mounted to an articulating frame and secured to a height-adjustable table. Participants performed maneuvers standing (except for French horn, for which participants sat in a chair), with the angle and height of the sampling inlet cone adjusted so that their instrument bell was positioned directly in front of and in approximate planar alignment with the center of the cone face (in the case of singers, the inlet cone was adjusted to align with their mouth). An isokinetic sample probe (0.05 m inner diameter) was installed at the narrow end of the inlet cone (0.22 m from the front plane of the cone face). Connected to the probe was an optical particle counter (OPC; model 11D, GRIMM Tech.) used to quantify the number and size of particles between 0.25 and 35.15 μm in diameter (31 logarithmically spaced size bins at five-second resolution; inlet flow 1.2 L min^−1^). Carbon dioxide (CO_2_) mixing ratios were measured further downstream at 1-s resolution with a non-dispersive infrared spectrometer (LI-820, LI-COR Biosciences). Outflow from the sampling apparatus was exhausted to the outdoors.

In a subset of performers, we also measured sound pressure levels (n = 32 participants) and the effect of bell covers (n = 67 participants) on reducing aerosol emissions from instruments. Sound pressure levels (i.e., instrument loudness above ambient background) were recorded during the maneuvers at a fixed location approximately 0.3 m above the face of the sampling cone using a prepolarized free-field condenser microphone with a preamplifier (Model 378B02l + 426E01, PCB Piezotronics Inc.). Bell covers were constructed from two layers of spandex and an inner layer of Halyard H600 medical wrap and sized to fit a variety of instrument bells. For particles larger than 1 μm in aerodynamic diameter, the efficiency of the bell cover was 95–99.9% (see supplement for details on aerosol collection efficiency of bell covers). Efficiency decreased to approximately 80% at 0.5 μm (the lower size limit for this protocol). Not all participants opted to use the bell covers due to poor sizing/fit (i.e., available covers did not fit all instruments); a few participants elected not to use the bell cover due to perceived airflow restrictions and/or impeded instrument playability. Bell cover results were therefore restricted to instruments with 3 or more participant measures to provide a measure of statistical confidence.

To establish background levels, participants were asked to sit in a corner of the chamber, approximately 2 m from the sampling apparatus, while wearing a face mask. For each background measurement, participants waited, prior to and after each *maneuver,* until total particle counts approached 50 L^−1^, as determined by the OPC. During this time, background data were also recorded for CO_2_ and ambient sound pressure levels. Further details on instrumentation, the measurement system, and background corrections are provided in the online supplement.

### Data analyses

All data analyses were conducted in R (R Core Team, version 4.1.2, https://www.r-project.org/). Time-series data for each participant were averaged across the maneuver (each ~ 4-min duration). Results are reported in terms of *near-field concentration* (i.e., particle concentration measured in the sampling apparatus immediately downstream of the instrument [particles L^−1^ of sampled air]), *particle emission rate* (i.e., particles s^−1^), or CO_2_-normalized *emission factor* (i.e., near-field concentrations divided by the measured CO_2_ mixing ratio, corrected for background).

We developed linear mixed models to explore how the following variables affect aerosol emissions: instrument type and/or class (i.e., vocal, woodwind, brass), maneuver type (scales, selection, freestyle), instrument sound pressure level (A-weighted decibels), participant sex (assigned at birth; male vs. female), participant age (minor vs. adult), and the use of a bell cover. The models evaluated maneuver, sex, age, sound pressure level, and instrument type (or class) as fixed effects, including a random intercept term to account for correlation within repeated measures from each participant. Measured emissions data were natural-log transformed to reduce skewness in their distribution. Results are reported as percent change in geometric mean emissions relative to a given fixed effect, along with 95% confidence intervals (CI). The linear mixed models take the following general form:

1 $$Y_{i,j} = \ln \left( {emission rate_{i,j} } \right) = {\upbeta }_{0} + {{\varvec{\upbeta}}}^{T} {\varvec{X}}_{i,j} + \alpha_{i} + \epsilon_{i,j}$$

where ${Y}_{i,j}$ represents the log-transformed emissions for the *i*th participant and *j*th maneuver, ${{\varvec{X}}}_{i,j}$ represents a set of fixed-effect variables (e.g., age, sex, class of instrument, etc.) for each measurement, ${\varvec{\upbeta}}$ is the vector of coefficients for the fixed effects, and ${\alpha }_{i}$ represents a random intercept term for participant *i*. The last term, ${\epsilon }_{i,j}$, represents residual model error (i.e., unexplained variation) which is assumed to have a mean of zero and be normally distributed with constant error variance. We used a likelihood ratio test to assess whether there was a significant difference in geometric mean aerosol concentration between different maneuvers, sexes, or ages. Statistical significance was assessed at the 0.05 level. Percent variation explained by the model was calculated using conditional and marginal R^2^^39^.

The relationship between sound pressure level and emissions was assessed with Pearson correlations and a linear mixed model that included fixed effects for sound pressure level and its interaction with instrument type. The effect of bell covers was evaluated through use of an interaction term with each instrument in a model that also controlled for age, sex, and participant. For the bell cover analyses, we normalize emissions data to the background-corrected CO_2_ mixing ratio measured downstream of the instrument, as prior work suggests that barriers and/or loosely fitted control technologies may redirect the flow of air orthogonal to the direction of exhaled air^8,40,41^. Thus, by normalizing to CO_2_ we account for sampling losses (i.e., air that did not enter the sampling apparatus) in measured emissions that could differentially bias comparisons with vs. without a bell cover. We tested the effects of CO_2_ normalization in subsequent sensitivity analyses.

## Supplementary Information

Supplementary Information.

## Data Availability

The summary dataset used to analyze and generate results for the current study is available in the Mountain Scholar open-access data repository https://hdl.handle.net/10217/235366.
