# Supplementary material for: Aerosol emissions from wind instruments: effects of performer age, sex, sound pressure level, and bell covers
Source: Sci Rep. 2022 Jul 4;12:11303. doi: 10.1038/s41598-022-15530-x (PMC9252563; doi:10.1038/s41598-022-15530-x)
Supplement: Supplementary file 1 — Supplementary Information. [file 41598_2022_15530_MOESM1_ESM.pdf]

Supporting Information:

Aerosol Emission Factors from Wind Instruments: Effects of Performer Age, Sex, Sound Pressure Level, and Bell Covers

Authors: *John Volckens*, Kristen M. Good, Dan Goble, Nicholas Good, Josh Keller, Amy Keisling, Christian L'Orange, Emily Morton, Rebecca Phillips, and Ky Tanner*

** Corresponding author, John.Volckens@colostate.edu*

<u>*Test Facility and Sampling Setup*</u>

The test chamber is ventilated with two independent systems that direct air flow into and out of the chamber while maintaining chamber pressure at near-ambient levels. HEPA-filtered air entered the chamber through a series of evenly spaced, perforated ducts across the ceiling. Air exited through a similar ducting arrangement (mirroring the ceiling) that was mounted under the chamber's permeable floor and exhausted to the outdoors. The air change rate in the chamber was set to 8.5 hr^{-1}. Participants entered the chamber through a two-door anteroom to minimize the infiltration of outdoor particles. After entering, participants sat for ~10min, while wearing their personal face covering, to allow any infiltrated aerosol to clear out, prior to initiating their maneuvers. Large windows were present on two sides of the chamber and an intercom system was installed so that study staff could monitor and communicate with participants regularly. A schematic and image of the sampling apparatus, located within the chamber, is shown in Figure S1.

In addition to OPC measurements (particle sizes from 0.25 to 35.15 μm), a condensation particle (CPC; Model 3789, TSI Inc., MN) was used to measure number of particles greater than 7nm in diameter (recorded single particle data at greater than one-second resolution; inlet flow rate 0.6 $L\cdot min^{-1}$) and a scanning mobility particle sizer (SMPS; Model 3938 [DMA 3081], TSI Inc., MN), was configured to measure the size distribution of particles between 10 and 500 nm (60-second resolution, flow rate of 0.6 $L\cdot min^{-1}$). *Note: SMPS and CPC data are not presented here because the size ranges reported by these instruments overlap with that of the OPC and particles below 0.25μm are less likely to carry SARS-CoV-2 virions.* Make-up air was drawn through one side of the T-fitting connected to the end of the cone to bring the total airflow through the cone to 10 $L\cdot min^{-1}$, to roughly match the ventilation rates of the participants. A flow-straightening gasket was inserted downstream of the three sample probes.

Sound pressure levels were recorded during the maneuvers at a fixed location approximately 30 cm above the face of the sampling cone using a prepolarized free-field condenser microphone with a preamplifier (Model 378B02l + 426E01, PCB Piezotronics Inc.). Frequency data were A-weighted, background corrected, and time-averaged for each maneuver.

The *near-field aerosol concentration* emitted by each instrument was calculated by adjusting the particle total concentration measured by the OPC in the sampling cone for the background particle concentration. The total background particle concentration was calculated from the one-minute of OPC data measured during the break (while the participant was sitting, masked, away from the inlet) prior to each maneuver. The *aerosol emission rate* for each participant was calculated by multiplying the near-field concentration by the total flow of the sampling apparatus (10 $L\cdot min^{-1}$).

The *raw concentration* limit of quantification was estimated based on the OPC counting one particle per six-second sampling interval above background, which given the sample flow rate of 1.2 $L\cdot min^{-1}$, corresponds to 50 particles per liter in the lowest size bin. This limit corresponds to an emission rate limit of 8.3 $particles\cdot s^{-1}$.

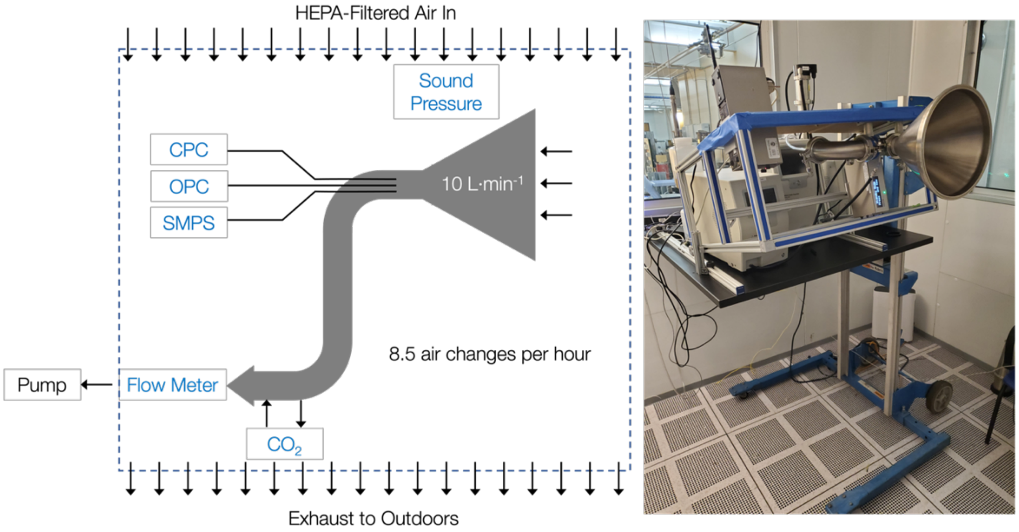

Figure S1: Sampling Setup. Left: Measurement schematic (not to scale) of the sampling cone, flow apparatus, and associated measurement instrumentation. Right: Image of the setup mounted to a height-adjustable table and articulating inlet within the cleanroom environment

__Participant Maneuvers__

The *selection* pieces were chosen by music faculty on study's Scientific Advisory Board (see acknowledgements) and were targeted towards the ability level of beginner (middle school aged musician relatively new to their instrument), intermediate (high school aged musician with several years of experience), or advanced (college-aged, professional, or other adult musician, with several years of experience on their instrument). The chosen selections were intended to cover a representative range of notes and playing patterns that are typical for a given instrument. Participants were provided with the selection piece in advance of the study session and asked to familiarize themselves with it. This was to ensure they were comfortable with the ability level they were assigned to and were not seeing the selection for the first time during the study session; however, they were told that they did not need to have the piece perfected prior to the study session. In fact, it was encouraged, particularly for beginner/intermediate musicians, to play the piece as they would in a "practice" session, rather than a performance or audition, so that we could capture a realistic range of what happens when playing the instrument.

__Bell Cover Filtration Performance__

We tested the aerosol collection efficiency of the bell covers using the protocol described by Leith et al.[1] Briefly, the cover was tested in a sealed chamber containing an oil droplet aerosol at a fixed concentration. The collection efficiency (i.e., the percentage of particles flowing into the cover that are removed via filtration) was calculated by comparing particle counts read by an aerodynamic particle sizer (TSI model 3321) when the cover was attached to the instrument to particle counts read when the mask was not attached to the instrument. Air was pulled through the cover at 15 liters per minute. For particles larger than 1 μm in aerodynamic diameter, the efficiency was 95-99.9%. Efficiency decreased to approximately 80% at 0.5 μm (the lower size limit for this protocol). These results are consistent with published reports for masks made from similar material.[2] Note: these results only depict the filtration performance of the mask material under ideal (i.e., non-leaking) conditions.

Participant Demographics

A histogram of participant ages at the time of enrollment (stratified by sex) is shown in Figure S2.

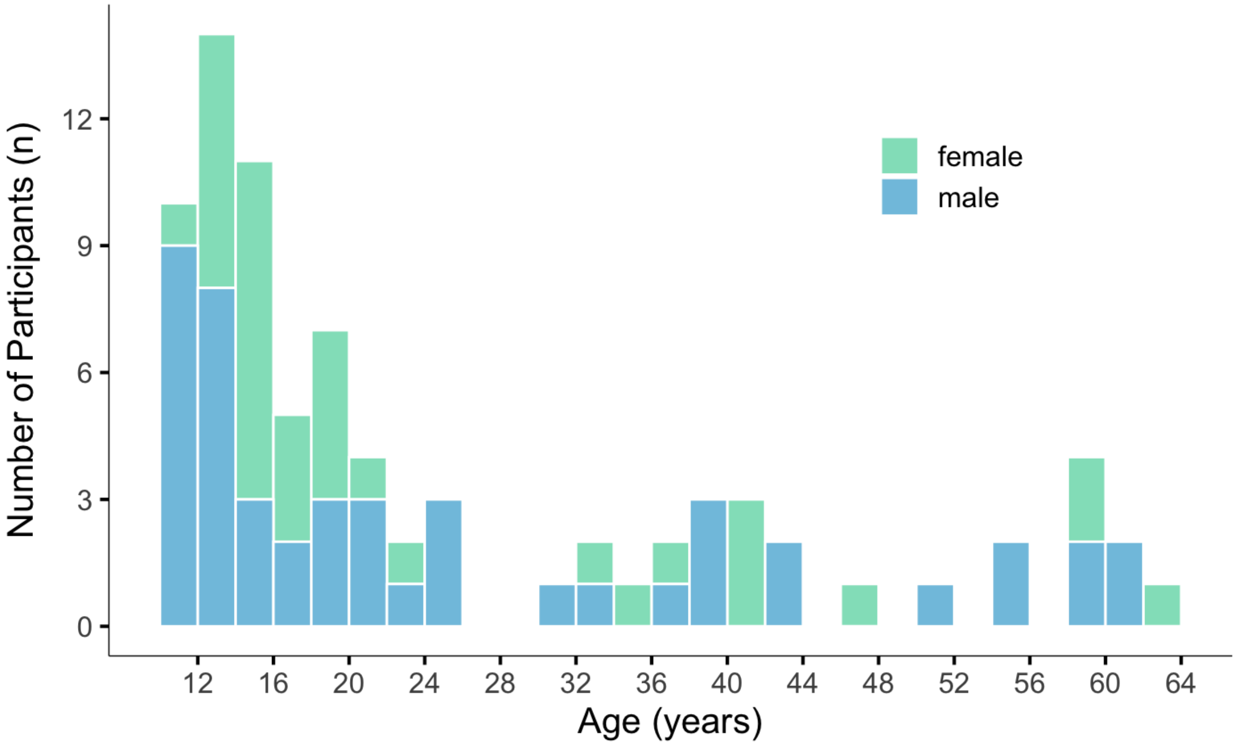

Figure S2: Histogram of participants by age and sex assigned at birth.

Within- and Between-participant Variation in Emissions

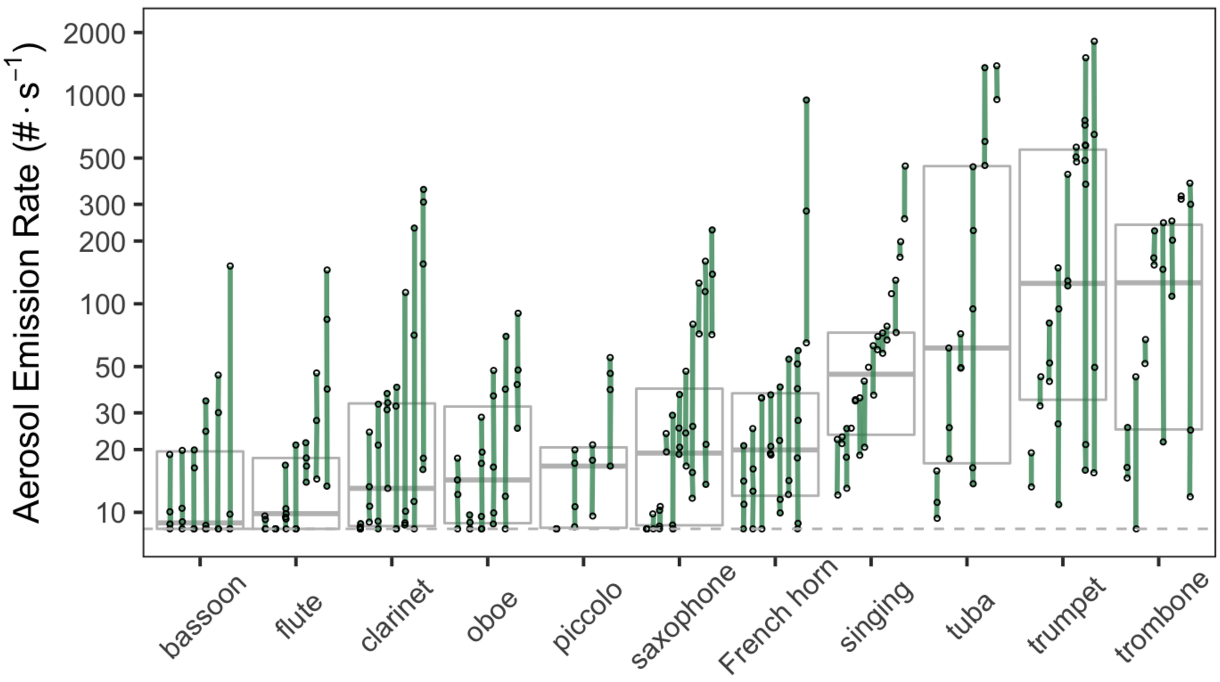

Figure S3: Boxplots of aerosol number emission rates (0.25 – 35.15 μm size range) by instrument type and participant. Instruments are ordered by median emission rate. Data for each participant are shown as a vertical line with individual measures as open circles. Boxes delineate the median and inter-quartile range (IQR) across all observations for a given instrument. The dashed horizontal line represents a method quantification limit (8.3 particles per second). All data are background corrected.

Aerosol Emission Rates by Instrument Class

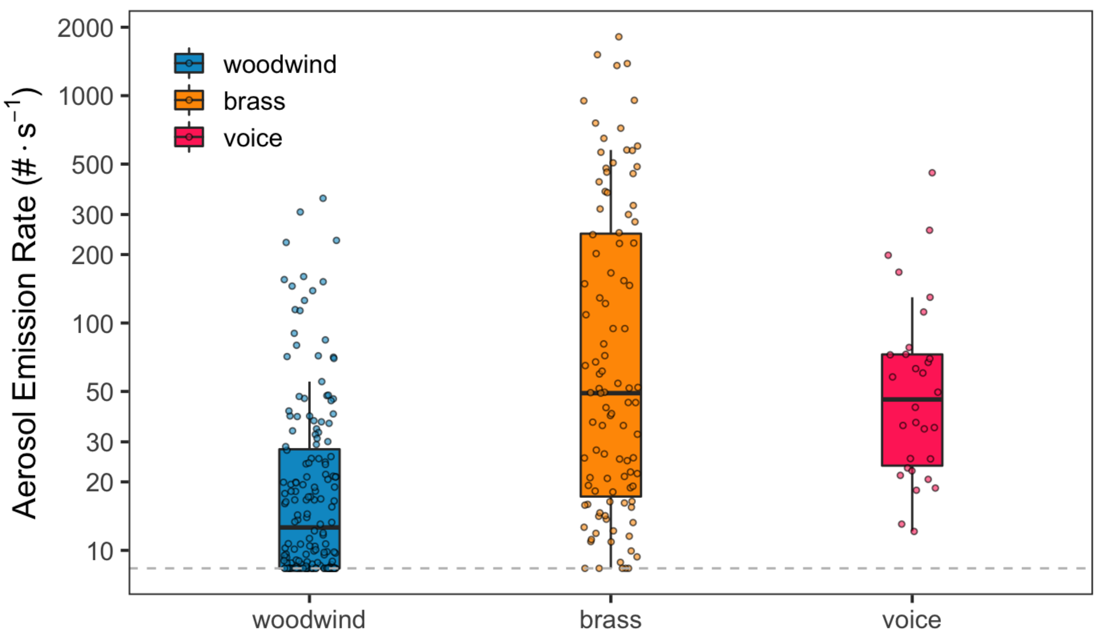

Figure S4: Boxplots of aerosol number emission rates (0.25 - 35.15 μm size range) by instrument class. Box centerlines represent median values, box limits delineate the inter-quartile range (IQR), and whiskers extend 1.5·IQR or the data minimum. Individual participant measures are shown as open circles. The dashed horizontal line represents a method quantification limit (8.3 particles per second). All data are background corrected.

Aerosol Emission Rates by Instrument Class and Age

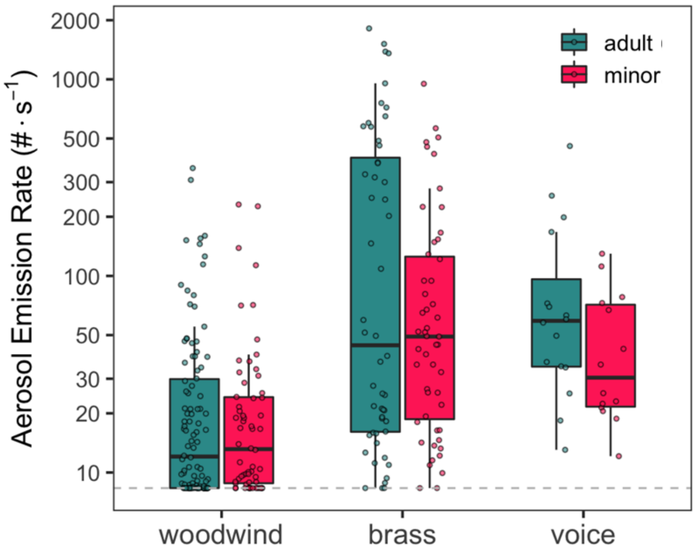

Figure S5: Boxplots of aerosol number emission rates (0.25 - 35.15 μm size range) by instrument class and participant age category. Box limits delineate the inter-quartile range (IQR) with median values at center; whiskers extend to 1.5·IQR or the data minimum. The dashed horizontal line represents a method quantification limit (8.3 particles per second). All data are background corrected.

Measured CO_2 Mixing Ratios by Instrument Class and Participant Sex

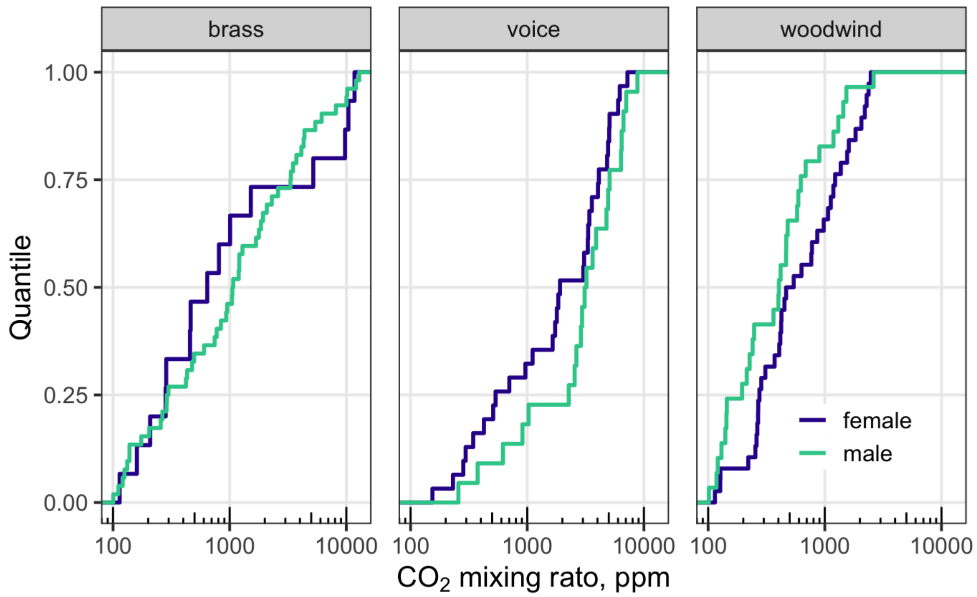

Figure S6. Cumulative distribution plots of measured CO_2 mixing ratios by instrument class and participant sex. Data represent mixing ratios above background and are restricted to values at least 20% above background.

Aerosol Size Distributions by Instrument Type

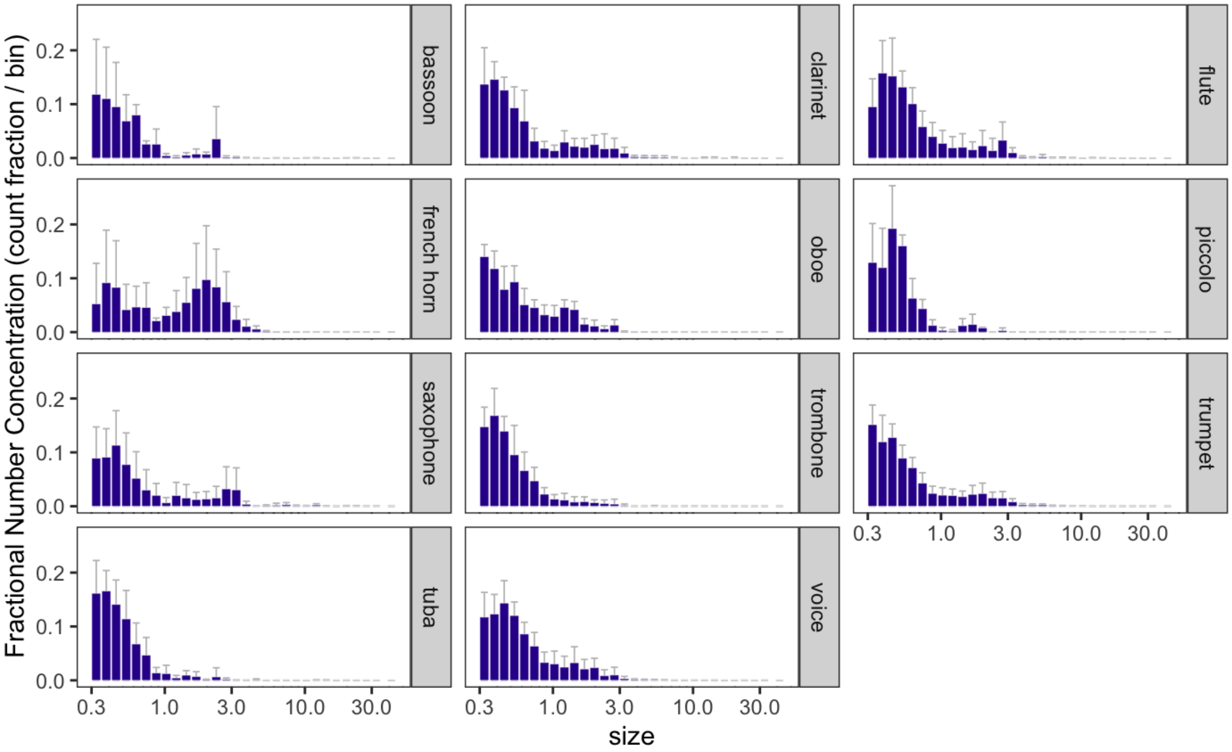

Figure S7: Average aerosol size distributions (count fraction) by instrument type (0.25 - 35.15 µm size range). Note: count fractions are not normalized to the logarithm of the bin width because all bin widths are equivalent (in log space). Data are background corrected and include maneuvers for which the count total exceeded method quantification limits (50 particles·L^{-1}). Error bars represent 1 SD about the mean.

Effect of Bell Covers on Measured CO_2 Mixing Ratio by Instrument Class

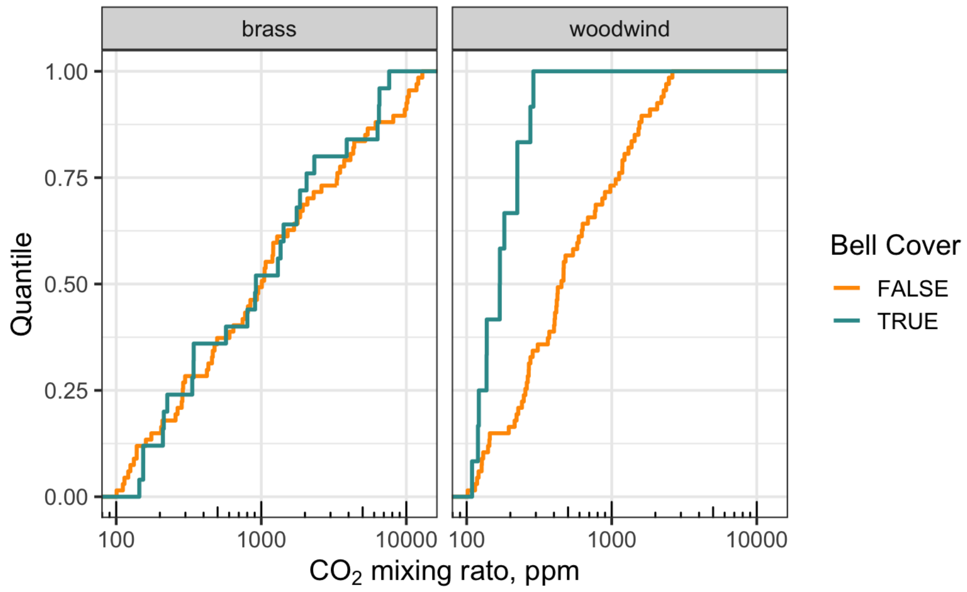

Figure S8. Cumulative distribution plots of measured CO_2 mixing ratio for brass and woodwind instruments played with and without bell covers. Data represent mixing ratios above background and are restricted to values at least 20% above background.

Sensitivity Analysis: Bell Cover Effect with and without Normalization to CO_2 Emissions

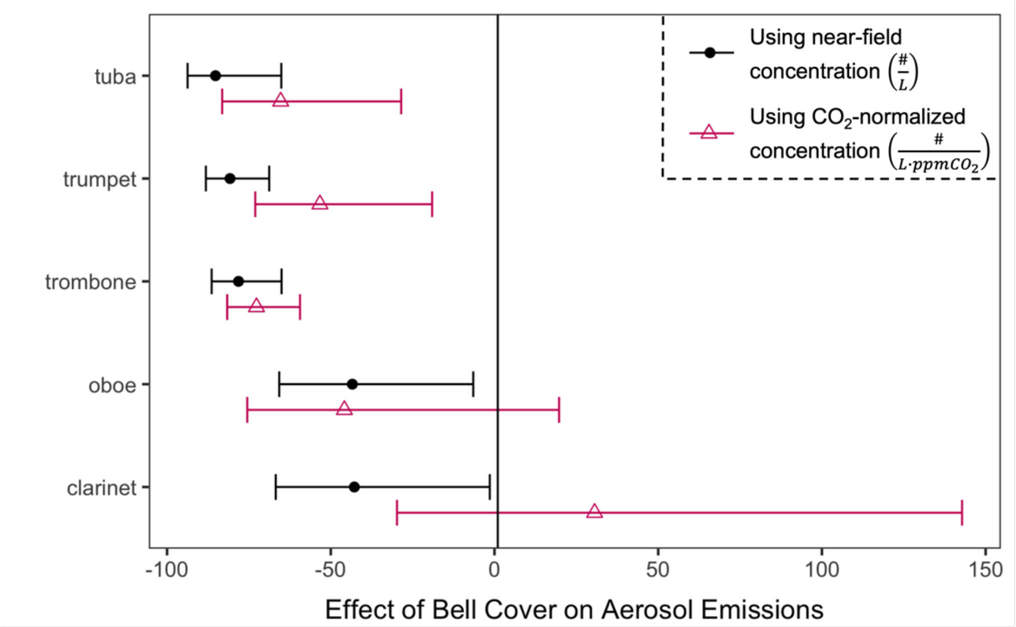

Figure S9. Comparison of bell cover effectiveness on reducing aerosol number emissions when using near-field concentrations (black line) vs. CO_2-normalized concentrations (magenta line). Points indicate average percent reduction in emissions, controlling for participant, with 95% confidence intervals. Data are background corrected and restricted to instruments with n ≥ 3 measurement pairs.

Descriptive Data Tables

Table S1. Near-field Aerosol Concentrations (particles·L^{-1}) by Instrument Type									
Instrument	Geometric Mean	GSD	Mean	SD	Min	Median	Max	IQR	n
bassoon	80.4	2.1	117.6	172.0	<50.0	53.4	911.2	67.6	26
flute	87.1	2.1	128.3	172.1	<50.0	59.1	871.8	59.4	29
clarinet	125.8	3.1	290.8	507.6	<50.0	78.2	2121.6	148.1	35
oboe	104.3	2.1	140.6	125.5	<50.0	85.8	540.5	141.2	27
piccolo	94.4	1.9	118.1	90.5	<50.0	100.0	331.4	72.4	15
saxophone	136.4	2.8	242.1	309.7	<50.0	115.4	1356.8	184.7	36
French horn	146.2	2.8	367.8	1013.4	<50.0	119.6	5695.9	151.4	32
singing	295.8	2.4	459.2	553.4	72.7	276.6	2747.7	295.0	30
tuba	527.7	5.7	1854.2	2723.1	56.2	368.3	8307.8	2641.6	19
trumpet	788.8	4.8	2070.7	2642.8	65.4	752.1	10891.2	3087.2	30
trombone	478.1	3.5	847.4	732.3	<50.0	765.2	2273.8	1287.4	22
Number concentrations represent total counts for particle sizes from 0.25 to 35.15 μm. Data are background corrected. GSD: Geometric Standard Deviation; SD: Standard Deviation; IQR: Interquartile Range; n: total number of participant maneuvers recorded. Values below the method detection limit (50 L^{-1}) are reported as <50.0.									

Table S2. Aerosol Number Emission Rates (particles·s^{-1}) by Instrument Type									
Instrument	Geometric Mean	GSD	Mean	SD	Min	Median	Max	IQR	n
bassoon	13.4	2.1	19.6	28.7	<8.3	8.9	151.9	11.3	26
flute	14.5	2.1	21.4	28.7	<8.3	9.9	145.3	9.9	29
clarinet	21.0	3.1	48.5	84.6	<8.3	13.0	353.6	24.7	35
oboe	17.4	2.1	23.4	20.9	<8.3	14.3	90.1	23.5	27
piccolo	15.7	1.9	19.7	15.1	<8.3	16.7	55.2	12.1	15
saxophone	22.7	2.8	40.4	51.6	<8.3	19.2	226.1	30.8	36
French horn	24.4	2.8	61.3	168.9	<8.3	19.9	949.3	25.2	32
singing	49.3	2.4	76.5	92.2	12.1	46.1	457.9	49.2	30
tuba	88.0	5.7	309.0	453.9	9.4	61.4	1384.6	440.3	19
trumpet	131.5	4.8	345.1	440.5	10.9	125.4	1815.2	514.5	30
trombone	79.7	3.5	141.2	122.0	<8.3	127.5	379.0	214.6	22
Number emission rates represent total counts for particle sizes from 0.25 to 35.15 μm. Data are background corrected. GSD: Geometric Standard Deviation; SD: Standard Deviation; IQR: Interquartile Range; n: total number of participant maneuvers recorded. Values below the method detection limit (8.3 s^{-1}) are reported as <8.3.									

Table S3. CO_2-normalized Aerosol Concentrations (particles·L^{-1}·ppmCO_2^{-1}) by Instrument Type									
**Instrument**	**Geometric Mean**	**GSD**	**Mean**	**SD**	**Min**	**Median**	**Max**	**IQR**	**n**
bassoon	0.074	2.578	0.106	0.083	0.013	0.072	0.246	0.124	12
flute	0.093	2.202	0.124	0.107	0.043	0.076	0.331	0.114	8
clarinet	0.121	2.560	0.178	0.152	0.019	0.123	0.592	0.200	53
oboe	0.199	2.689	0.290	0.260	0.025	0.230	0.961	0.221	11
piccolo	0.320	2.233	0.464	0.572	0.082	0.279	2.602	0.198	18
saxophone	0.238	2.665	0.358	0.335	0.024	0.288	1.316	0.217	27
French horn	0.320	3.320	0.677	1.275	0.021	0.315	5.798	0.419	19
singing	0.373	2.052	0.485	0.404	0.132	0.327	1.400	0.339	16
tuba	0.386	2.630	0.570	0.509	0.029	0.332	2.183	0.615	31
trumpet	0.233	3.034	0.344	0.242	0.030	0.341	0.707	0.321	9
trombone	0.324	1.837	0.385	0.246	0.091	0.353	1.123	0.250	20
Number concentration factors represent total counts for particle sizes from 0.25 to 35.15 μm. Data are corrected for background aerosol and CO_2 levels and restricted to CO_2 measures exceeded background levels by > 20%. GSD: Geometric Standard Deviation; SD: Standard Deviation; IQR: Interquartile Range; n: total number of participant maneuvers recorded.									

Table S4. Aerosol Number Emission Rates (particles·s^{-1}) by Type of Music Played									
**Music Type**	**Geometric Mean**	**GSD**	**Mean**	**SD**	**Min**	**Median**	**Max**	**IQR**	**n**
scales	28.9	3.7	96.6	246.6	<8.3	18.9	1815.2	40.3	118
self-selected repertoire	29.4	3.5	80.3	153.5	<8.3	21.0	719.2	48.1	74
prescribed repertoire	33.3	3.8	105.1	233.0	<8.3	23.0	1384.6	61.5	109
Emission rates represent particle sizes from 0.25 to 35.15 μm. Data are background corrected. GSD: Geometric Standard Deviation; SD: Standard Deviation; IQR: Interquartile Range; n: total number of participant maneuvers recorded. Values below the method detection limit (8.3 s^{-1}) are reported as <8.3.									

References Cited

1 Leith, D., L'Orange, C. & Volckens, J. Quantitative protection factors for common masks and face coverings. *Environmental Science & Technology* **55**, 3136-3143 (2021).

2 Sousan, S., Garcia, N., White, A. & Balanay, J. A. Filtration efficiency of surgical sterilization fabric for respiratory protection during COVID-19 pandemic. *American Journal of Infection Control* **49**, 1-7, doi:https://doi.org/10.1016/j.ajic.2020.11.005 (2021).
